# FDA approves neffy nasal spray for treatment of anaphylaxis in adult and pediatric patients

**DOI:** 10.1097/MS9.0000000000003510

**Published:** 2025-06-18

**Authors:** Zainab Syyeda Rahmat, Rhea Chandwani, Hamza Maqbool, Abdullah Malikzai

**Affiliations:** a Faculty of Medicine, Dow Medical College, Dow University of Health Sciences, Karachi, Pakistan; b Department of Medicine,Kasturba Medical College, Manipal, India; c Department of Medicine,Continental Medical College, Lahore, Pakistan; d Kabul University of Medical Sciences, Kabul, Afghanistan

## Introduction

On August 9, 2024, the U.S. Food and Drug Administration (FDA) approved neffy (ARS Pharmaceuticals, Inc, San Diego, Calif), a nasal spray containing epinephrine for the treatment of allergic reactions in an emergency setting^[1]^. Manufactured by ARS Pharmaceuticals, neffy is a two-mg nasal spray for the treatment of type I allergic reactions, including anaphylaxis, in adults and children who weigh 30 kg (66 lbs) or above^[2]^. Neffy represents the first significant innovation in the delivery of epinephrine in more than 35 years and is the first and only needle-free treatment option for patients and families living with severe allergic reactions^[1]^.

Allergic reactions are abnormal immune responses to any substance that is generally well-tolerated by others, and anaphylaxis is a life-threatening allergic reaction that can manifest within minutes as a rapidly progressive generalized or systemic hypersensitivity reaction and can result in death in less than 15 minutes^[1,3].^ Medication allergies have also been documented to cause death in an average of 5 minutes^[3]^. Anaphylaxis is a global health concern with the incidence increasing over the past decades, and the current incidence rate is approximately 46 cases per 100 000 population per year^[4]^. In the United States, between 1.6% and 5.1% of the general population has experienced an anaphylactic episode at least once^[3]^. Common precipitants of anaphylaxis include food, latex, insect venom, and certain medications^[3]^. As it can result in potentially fatal respiratory failure, circulatory collapse, and cardiac arrest, anaphylaxis is considered a medical emergency.

International guidelines state that the first-line in-hospital treatment option for anaphylaxis is intramuscular (IM) epinephrine, and patients are usually prescribed epinephrine auto-injectors (EAIs) at discharge^[5,6]^. Epinephrine itself is the only life-sustaining treatment option for anaphylactic reactions^[3]^. Other second-line drugs include antihistamines, glucocorticoids, and beta-adrenergic agonists^[5]^. While the existing treatment regimen is effective, the current first-line IM route of epinephrine administration outside of hospital settings has been proven in studies to be cumbersome to use in emergencies^[5]^. In a study of over 1000 pediatric patients, 56% of their parents displayed fear regarding the correct use of EAIs and distress over causing harm to the child^[7]^. Even if caregivers carry EAIs, up to 83% either do not administer or delay the adminstration of EAIs in emergency situations^[8]^.

Various other challenges met while administering EAIs include doubts of correct administration, fear of needles, inadequate training, and inflated costs^[2,7,8]^. Neffy addresses these issues by offering an easy-to-use, needle-free alternative that can reduce treatment delays and potentially save additional lives^[8]^. It exhibits a similar pharmacodynamic and pharmacokinetic profile to that of EAIs and is well tolerated^[8]^.

## Methods

Articles on PubMed were searched for clinical trials related to neffy and were thoroughly reviewed until August 2024. Information deemed important and relevant was subsequently screened and included in this article. Our search strategy included keywords such as “neffy,” “epinephrine auto injectors,” and “anaphylaxis.” Literature unrelated to neffy, EAIs, or anaphylaxis, or which dated before 2011 was not included.

## Discussion

Manual administration of IM epinephrine in the hospital effectively reverses anaphylactic symptoms like bronchoconstriction and vasodilation when administered promptly^[9]^. However, although efficacious, using EAIs like EpiPens has proved challenging due to fears of incorrect administration, needle phobia, uncertainty about timing, and complexity of devices leading to potentially dangerous delays^[2,7]^. Occasional shortages and their expensive cost impede the widespread use of EAIs as well^[10]^. The amalgamation of these factors indicated the crucial need for a fast, reliable, and easily administered treatment for anaphylaxis. Therefore, neffy was given fast-track approval by the FDA to address the urgent need for effective anaphylaxis management outside hospital settings^[1]^.

A cardinal feature of anaphylaxis is a sudden drop in blood pressure, termed as circulatory shock, which may quickly lead to death^[3]^. As such, an adequate and sustained increased in blood pressure is vital in the resuscitation of a patient experiencing anaphylaxis. Based on an integrated analysis comparing epinephrine administration by IM route versus intranasally, neffy was found to increase systolic blood pressure comparably to the IM route, and was the only product that increased diastolic blood pressure in patients over time^[9]^. The safety of neffy is based on four clinical studies in 175 healthy adults without anaphylaxis, and concentrations of epinephrine in the patient’s blood were recorded after administering IM or intranasal epinephrine, finding the concentrations equal^[1]^. One randomized control trial revealed that neffy displayed more distinct pharmacodynamic parameters when dosed both once and twice when compared to Epipen or manual epinephrine injection^[8]^. Another study compared the stability of these epinephrine products under extreme temperature changes and found that neffy was the most stable product displaying the highest epinephrine potency in extreme temperatures, which depicts its viability unrelated to significant weather changes^[11]^. Evidence from these studies supports the use of neffy as it adequately tackles patient nonadherence and has the most adequate potency in hot weather conditions as compared to traditional products.

Administered to one nostril once, neffy addresses needle phobia and correct usage issues while providing a noninvasive substitute for IM epinephrine injections^[1]^. The spray’s easy-to-use design may increase adherence and accelerate emergency response times. Although neffy is still in its infancy and might not be as widely available as conventional auto-injectors, it could provide better options for anaphylaxis treatment, especially for adults and children with injection fears.

The most common adverse reactions of neffy, with an incidence of less than 2%, include throat irritation, headache, nasal discomfort, jitteriness, fatigue, and nasal congestion among others^[8]^.

Neffy comes with a warning that certain nasal conditions, such as nasal polyps or a history of nasal surgery, may affect absorption of neffy, and patients with these conditions should consult with a healthcare professional regarding alternatives. Neffy also comes with warnings and precautions about the use of epinephrine by people with certain coexisting conditions and allergic reactions to sulfite^[1]^.

Despite these findings, further research is necessary to fully assess the long-term safety and efficacy of neffy. The approval of this nasal spray was based on four clinical studies, but patients in these studies did not have anaphylactic symptoms when neffy was administered to them due to ethical and physical barriers^[12]^. Additionally, ongoing studies will need to address any potential gaps in the current evidence base, such as its performance in very young children or individuals with certain preexisting conditions.

A new era of research and groundbreaking discoveries is being brought forth by AI models such as the AlphaFold model, which can quickly produce optimum formulation as well as enhance the efficacy and safety of neffy and similar new innovations^[13]^. These AI models can identify high-risk populations for patients with allergic reactions, and for problems related to the stability of these medications at high temperatures, AI can provide predictions with accuracy^[14]^.

## Data Availability

Not Applicable.
